# Test-Retest Reliability of the Patient Activation Measure-13 in Adults with Substance Use Disorders and Schizophrenia Spectrum Disorders

**DOI:** 10.3390/ijerph18031185

**Published:** 2021-01-29

**Authors:** Katrine Melby, Mona Nygård, Mathias Forsberg Brobakken, Rolf W. Gråwe, Ismail Cüneyt Güzey, Solveig Klæbo Reitan, Einar Vedul-Kjelsås, Jørn Heggelund, Paul B. Tchounwou

**Affiliations:** 1 Department of Clinical and Molecular Medicine, Norwegian University of Science and Technology (NTNU), 7491 Trondheim, Norway; katrine.melby@stolav.no; 2 Blue Cross Lade Addiction Treatment Centre, 7041 Trondheim, Norway; 3 Department of Clinical Pharmacology, St. Olavs Hospital, Trondheim University Hospital, 7006 Trondheim, Norway; 4 Department of Mental Health, Norwegian University of Science and Technology (NTNU), 7491 Trondheim, Norway; mathias.f.brobakken@ntnu.no (M.F.B.); rolf.w.grawe@stolav.no (R.W.G.); cuneyt.guzey@ntnu.no (I.C.G.); Solveig.Klebo.Reitan@stolav.no (S.K.R.); einar.vedul-kjelsas@ntnu.no (E.V.-K.); mariela.lara@ntnu.no (M.L.L.-C.); 5 Department of Østmarka, Division of Mental Health Care, St. Olavs Hospital, Trondheim University Hospital, 7006 Trondheim, Norway; 6 Department of Research and Development, Division of Mental Health Care, St. Olavs Hospital, Trondheim University Hospital, 7006 Trondheim, Norway; 7 Regional Center for Healthcare Improvement, St. Olavs Hospital, Trondheim University Hospital, 7006 Trondheim, Norway; jorn.heggelund@stolav.no; 8 Tiller Community Mental Health Centre, Division of Psychiatry, St. Olavs Hospital, Trondheim University Hospital, 7006 Trondheim, Norway

**Keywords:** patient activation measure-13, internal consistency, test–retest reliability, self-management, schizophrenia spectrum disorders, substance use disorder

## Abstract

Patient Activation Measure-13 (PAM-13) is a valid and widely used questionnaire that assess an individual’s knowledge, confidence, and skills for self-management of their chronic illness. Although there is some evidence regarding its reliability, the test–retest reliability has not been investigated among patients with substance use disorders (SUDs) or schizophrenia spectrum disorders. We investigated the internal consistency and test–retest reliability of PAM-13 in these populations. Test–retest reliability was analysed using data from 29 patients with SUDs and 28 with schizophrenia spectrum disorders. Cronbach’s α and Intraclass Correlation Coefficient (ICC) scores were used to examine internal consistency and test–retest reliability, respectively. Of the 60 collected test–retest questionnaires, 57 were included in the analyses. No mean differences between time one (T1) and time two (T2) were observed in either patient group, except for item 12 in schizophrenia spectrum disorders patients (*p* < 0.05). Internal consistency for T1 and T2 was 0.75 and 0.84 in SUDs patients and 0.87 and 0.81 in schizophrenia spectrum disorders patients, respectively. The ICC was r = 0.86 in patients with SUDs and r = 0.93 in patients with schizophrenia spectrum disorders. To conclude, PAM-13 showed good internal consistency and test–retest reliability in SUDs and schizophrenia spectrum disorders patients.

## 1. Introduction

Substance use disorders (SUDs) and schizophrenia spectrum disorders often require long-term treatment due to early onset, long-lasting and severe symptoms [1,2]. Both patient groups commonly have relapses with detrimental effects for recovery [3,4,5]. Patients also engage in detrimental lifestyles that result in poor health status and shortened life expectancy [6]. Patient activation can be defined as the knowledge, confidence, and skills for self-management of one’s health and health care. Patient activation emphasizes the patients’ willingness and ability to take independent actions to manage their own health. As such, patient activation underlines the importance of understanding the patient’s own role in their care process. Having knowledge about one’s own treatment and knowing how to manage their condition, while remaining a functional member of society, is thus important. Accordingly, patients who have high activation levels believe they have important roles to play in self-management of their own care and tend to also have the confidence to collaborate with health-care providers [7].

Recent studies have indicated that patients with high patient activation, have better health status [8]. Other studies also suggest that patients having high activation levels have the skills and behavioural repertoire to manage their condition, even when under stress, since they know how to access to appropriate health care [7]. There is also a growing number of studies indicating that those who are more activated have better outcomes [7], and better use of health services [9]. They are more likely to adhere to treatment and take part in preventative behaviours. These positive behaviours include regular medical appointments, regular exercise, and a healthy diet. However, they are also more likely to avoid negative behaviours damaging their health, such as smoking and drug use. Additionally, they more often have prepared questions for doctor’s appointments, seeking information regarding treatment options for their illness and quality of health care [7]. Therefore, it is clearly necessary to have reliable instruments to capture patient activation.

Several self-reported questionnaires have been developed to measure patient activation defined as an individual’s knowledge, confidence, and skills for self-management of their chronic illness behaviour. In 2004, the 22-item Patient Activation Measure (PAM) was developed [7]. In 2005, Hibbard et al. [10] developed the 13-item version of PAM (PAM-13). The PAM-13 is the most used questionnaire developed to assess patient activation [8]. Research over the last decade has reported a positive relationship between high PAM-13 scores and better patient satisfaction and health outcomes [8]. Low PAM-13 scores were associated with higher rates of hospitalization and use of emergency room services among chronically ill patient populations [8,9,11].

PAM-13 ascertains the patient’s self-reported knowledge, skills and confidence in the self-management of his or her own health [10]. The questionnaire has been translated into several languages and validated for a range of mental and somatic illnesses [12,13,14,15,16,17,18,19]. PAM-13 has recently been used in studies that included patients with schizophrenia spectrum disorders [20,21,22,23,24], as well as patients with SUDs [25]. However, studies providing information about the test–retest reliability of the questionnaire in these settings are scarce. Test–retest reliability gives information regarding the extent to which two measurements, using the same questionnaire over a short period of time, remain the same when assessing the same unchanged individual [26]. In health research settings, the lack of test–retest reliability is an important methodological challenge, as it hinders reliable replication of results and conclusions. From clinical experience, this measurement property is also important, especially among patient groups that often are under the influence of medications and their side effects, or struggle with low confidence or poor self-esteem.

To our knowledge, six studies have investigated test–retest reliability [12,13,14,15,16,17]; of those, only one was conducted in mental health patients [14]. Although that study found PAM-13 to have good psychometric properties, mainly among outpatients with depression and anxiety disorders, the test–retest sample included neither patients with SUDs nor patients with schizophrenia spectrum disorders. People with substance use disorders and patients with schizophrenia spectrum disorders can have unstable medical conditions, and in difficult periods they can show a lack of insight into their own health situation. Reliable self-reported questionnaires, validated in clinical settings, are important to identify and plan medical treatments, according to the patient activation needs. Ensuring test reliability in these settings is therefore important, therefore, this study aims to investigate the internal consistency of PAM-13 and the test–retest reliability estimates for patients with SUDs and patients with schizophrenia spectrum disorders.

## 2. Materials and Methods

### 2.1. Participants and Study Design

Data was retrieved from two studies conducted in Norway between 2016 and 2018, including outpatients with SUDs and schizophrenia spectrum disorders. Patient characteristics and clinical data are presented in Table 1.

### 2.2. Recruitment, Procedures and Data Collection

Assessments were conducted using the same in-person procedures at baseline (test, T1) and the following day (re-test, T2). Sociodemographic information was self-reported, and ICD-10 diagnoses were verified against administrative systems (Table 1).

### 2.3. Measures

The Norwegian version of PAM-13 was used [14] to assess the patient groups’ activation regarding their physical health. The questionnaire consists of 13 items which can be scored from (1) ‘strongly disagree’ to (4) ‘strongly agree’ or (0) ‘not applicable’. The total score is calculated by dividing the raw score by the number of answered items and multiplying it by 13. This score is further transformed through calibration tables, ranging from 0 to 100, with higher PAM scores indicating higher patient activation. Items are presented in Table 2.

### 2.4. Sample Size Estimation and Statistical Analyses

Since the test–retest reliability involved two observations, a minimum of 22 participants was required to detect the value of 0.50 for the ICC [26]. To account for dropouts, 30 participants were recruited for each group. Data was analysed using SPSS, version 22 (IBM, Armonk, New York, NY, USA). Cronbach’s *α* was used to examine internal consistency. To assess test–retest reliability, ICC was calculated based on a two-way random-effects model. The mean differences between individual scores at T1 to T2 were calculated using the Wilcoxon signed-rank test and the paired *t*-test. Statistical significance was accepted at *p* < 0.05. All analyses were carried out separately for the two groups.

## 3. Results

Of the 78 patients invited to participate, 60 completed the baseline measures (T1). Subsequently, 29 SUDs and 28 schizophrenia spectrum disorders patients answered the re-test questionnaires. Data collection was performed between 2016 and 2018.

In SUDs patients, the mean item scores ranged from 2.55 (item 13) to 3.90 (item 2). There were no mean differences between T1 and T2, except for a trend in item 4 (*p* = 0.058, Table 2). ICC was *r* = 0.86, and Cronbach’s *α* was 0.75 and 0.84 at T1 and T2, respectively (Table 3). In schizophrenia spectrum disorders patients, the mean item scores ranged from 2.59 (item 13) to 3.52 (item 1). There were no mean differences between T1 and T2, except for item 12 (*p* < 0.05, Table 2) and a trend in item 4 (*p* = 0.052, Table 2). ICC was *r* = 0.93, and Cronbach’s *α* was 0.87 and 0.81 at T1 and T2, respectively (Table 3).

## 4. Discussion

Our findings show that, for SUDs patients and schizophrenia spectrum disorders patients, PAM-13 possesses good internal consistency, with Cronbach’s *α* values ranging between 0.75 and 0.87 at both assessments [27]. The Cronbach’s *α* values are comparable to those obtained in patient groups with chronic somatic illnesses [13,15,16,17,28], those undergoing surgery [12] and those with non-severe mental disorders [14].

Our ICC scores imply good test–retest reliability (≥0.70) [27,29] in both patient groups, reflected by only item 12 being significantly different at re-test among schizophrenia spectrum disorders patients, and a trend towards a difference for item 4 in both patient groups. To our knowledge, only one previous study investigated the test–retest reliability of PAM-13 in a mental health population [14]. As that study did not include patients with schizophrenia spectrum disorders or SUDs, our findings add to the limited information regarding the test–retest reliability of PAM-13 in a mental health setting. The ICC score for the patients with schizophrenia spectrum disorders was higher than what has been observed previously in outpatients with less severe mental disorders at one-month follow-ups [14], patients undergoing spine surgery at one-week follow-ups [12] or those with chronic diseases at two-week follow-ups [17]. ICC scores for SUDs patients were, however, comparable to the aforementioned studies.

It is interesting that the poor physical health, which has been observed previously in both SUDs and schizophrenia spectrum disorders patients [30,31,32,33,34], is also reflected in PAM-13, as both groups scored lowest on item 13, suggesting poor confidence in their abilities to maintain lifestyle changes regarding diet and exercise. As shown in this study, the measurement of patient activation may be helpful in clinical research, as well as a useful tool to identify patients in need of increased support regarding their own health management, which is often the case in these patient groups. Thus, PAM-13 can potentially contribute to improved patient involvement in their own health management.

The mean activation scores for our SUDs patients are comparable to what has previously been found in other SUDs patients [25], while the mean activation scores reported in mental disorder patients seem more spread, with our values of ~60 lying above most other reported values [14,20,23,24] but also lower than others [25].

Patient groups within mental health settings often struggle with several severe physical health challenges [30,31,32,33,34]. Thus, a strength of the current study is the use of PAM-13, the gold standard for assessing patient health activation. This study in turn, since it has evaluated the reliability of PAM-13 in a new setting, further expands the utilization of PAM-13 to understand the physical health challenges of these patient groups. A possible limitation to this study is the inclusion of patients from only two centres, which may limit the possibility of generalizing our findings. Another limitation could be the short test–re-test interval. However, as both patient groups are prone to relapse, having a long test–re-test interval would therefore not be ideal; nevertheless, it may be useful to consider replicating this study with a longer time interval. When planning reliability studies, it is recommended to have an a priori calculation of sample size [27,35]. Even though a sample size estimate for ICC was done a priori, there were no power calculation for internal consistency. According to methodological studies, a sample size of a minimum of 30 participants is an adequate estimator of the population coefficient alpha [36] as well as for ICC analysis [26]. Even though the aim was to include 30 participants in each sample, patients were lost to re-retest. This left less than 30 participants with complete test–retest data. This limitation must be considered when interpreting the results and planning future studies. To advance future research it is recommended to have an a priori calculation of sample size based on Cronbach’s alpha taking in consideration dropouts at retest. Further studies assessing principal component analysis, in a large sample, are recommended. Until this is done, the present study is an important step paving the way towards measuring patient activation and gaining knowledge regarding the reliability of PAM-13 among patients in addiction and mental health settings.

## 5. Conclusions

PAM-13 shows good internal consistency and test–retest reliability in adults with SUDs and schizophrenia spectrum disorders and may be helpful in a variety of clinical research settings, including inpatient and outpatient populations. For health care providers, PAM-13 can potentially be useful in identifying areas where different patients need increased support regarding their own health management, potentially resulting in less relapse and better activation.

## Figures and Tables

**Table 1:** Patient characteristics and clinical data for both patient groups.

	Substance Use Disorders (*N* = 29)	Schizophrenia Spectrum Disorders (*N* = 28)
Age, mean (*SD*)	46 (13)	34 (10)
Gender, male/female	18/11	17/11
*Primary ICD-10 Diagnosis, n*		
Mental and behavioural disorder due to		
Alcohol dependence (F10.2)	13	-
Use of opioids		
Opioid dependence (F11.2)	8	-
With opioid-induced mood disorder (F11.24)	1	-
With opioid-induced psychotic disorder (F11.25)	1	-
Cannabis use, unspecified (F12.9)	2	-
Sedative, hypnotic or anxiolytic-related dependence (F13.2)	1	-
Schizophrenia		
Paranoid (F20.0)	-	18
Undifferentiated (F20.3)	-	6
Residual (F20.5)	-	1
Schizoaffective disorder		
Manic type (F25.0)	-	2
Depressive type (25.1)	-	1
Comorbid Diagnoses (n) *		10
*Marital status, n*		
Married	8	2
Divorced	7	2
Not married/widow/widower	14	24
*Education, n*		
Primary	11	4
Secondary	13	22
Bachelor’s degree or higher	4	2
	1	
*Employment status, n*		
Student	-	1
Working	5	3
Sick leave	1	-
Rehabilitation	11	8
Disability pension	8	15
Retired	1	-
Unemployed	3	-
Working from home	-	1
Other	-	-
*Living with someone, n*		
Alone	18	15
Family	11	4
With others	-	-
Supportive housing	-	9

* Comorbid diagnoses (n): E11.9 (1), E66.8 (1), F10.1 (1), F15.2 (1), F19.2 (1), F40.1 (1), F60.9 (1), F63 (1), F84.5 (1), F90.0 (1), G40 (1), N39.4 (1).

**Table 2:** Statistics between mean scores at test and retest in both patient groups.

	Substance Use Disorders (*N* = 29)				Schizophrenia Spectrum Disorders (*N* = 28)			
	*n*	Mean T1 (*SD*)	Mean T2 (*SD*)	*p*	*n*	Mean T1 (*SD*)	Mean T2 (*SD*)	*p*
1. When all is said and done, I am the person who is responsible for managing my health condition	28	3.79 (0.42)	3.64 (0.68)	0.180	27	3.30 (0.78)	3.52 (0.58)	0.244
2. Taking an active role in my own health care is the most important factor in determining my health and ability to function	29	3.90 (0.31)	3.72 (0.65)	0.157	26	3.35 (0.85)	3.46 (0.58)	0.448
3. I am confident that I can take actions that will help prevent or minimize some symptoms or problems associated with my health condition	28	3.14 (0.76)	3.21 (0.63)	0.739	27	2.93 (0.78)	3.00 (0.73)	0.642
4. I know what each of my prescribed medications does	27	3.89 (0.32)	3.67 (0.62)	0.058	26	3.31 (0.74)	3.00 (0.75)	0.052
5. I am confident I can tell when I need to go get medical care and when I can handle a health problem myself	29	3.34 (0.55)	3.31 (0.60)	0.564	28	2.89 (0.57)	2.96 (0.51)	0.564
6. I am confident I can tell my health provider the concerns I have even when he or she does not ask	29	3.34 (0.81)	3.34 (0.77)	1.000	28	2.93 (0.90)	3.00 (0.67)	0.617
7. I am confident I can follow through on the medical treatment I need to do at home	28	3.21 (0.74)	3.32 (0.77)	0.257	24	3.33 (0.82)	3.46 (0.66)	0.564
8. I understand the nature and causes of my health condition	29	3.62 (0.56)	3.48 (0.69)	0.257	26	2.96 (0.87)	3.04 (0.66)	0.557
9. I know the different medical treatment options available for my health condition	29	3.31 (0.66)	3.21 (0.73)	0.417	26	2.77 (1.07)	3.08 (0.80)	0.153
10. I have been able to maintain the lifestyle changes I have made for my health	29	3.03 (0.87)	3.07 (0.75)	0.705	23	3.13 (0.87)	2.96 (0.98)	0.305
11. I know how to prevent further problems with my health condition	29	3.48 (0.69)	3.55 (0.57)	0.480	27	2.93 (0.87)	3.07 (0.78)	0.336
12. I am confident I can find a solution when new situations or problems arise with my health condition	29	3.00 (0.80)	3.00 (0.89)	1.000	27	3.30 (0.82)	3.04 (0.76)	0.035
13. I am confident I can maintain lifestyles changes, like diet and exercise, even during times of stress	29	2.62 (0.86)	2.55 (0.95)	0.739	27	2.70 (0.99)	2.59 (0.97)	0.477

Individual items response options ranged from 1–4. SD = standard deviation; T1 = test; T2 = retest.

**Table 3:** Reliability and internal consistency statistics.

	Substance Use Disorders (*N* = 29)	Schizophrenia Spectrum Disorders (*N* = 28)
Cronbach’s alpha T1	0.75	0.87
Cronbach’s alpha T2	0.84	0.81
Total mean (*SD*) T1	69.8 (11.6)	59.5 (15.0)
Total mean (*SD*) T2	67.6 (14.8)	60.2 (13.4)
ICC (95%CI)	0.86 (0.77–0.93)	0.93 (0.86–0.97)

SD = standard deviation; T1 = test; T2 = retest; ICC = intraclass correlation coefficient; CI = confidence interval.

## Data Availability

The data presented in this study are available from the corresponding author upon reasonable request. The data are not publicly available due to ethical reasons.
